# Breast cancer, prostaglandins and patient survival.

**DOI:** 10.1038/bjc.1989.56

**Published:** 1989-02

**Authors:** A. Bennett, I. F. Stamford, D. A. Berstock, F. Dische, L. Singh, R. P. A'Hern

**Affiliations:** Department of Surgery, King's College School of Medicine and Dentistry, Rayne Institute, London, UK.

## Abstract

Prostaglandins may have both undesirable and desirable effects in malignant disease. Their possible roles in breast cancer were studied by examining the relationships between different variables and the amounts of prostaglandin-like material (PG-LM) extracted from 141 breast carcinomas. Univariate analysis indicates a direct correlation with patient age and menopausal status, with a greater yield from cancers of post- compared with pre-menopausal women. Tumours up to 2 cm diameter yielded more PG-LM than those measuring greater than 2-5 cm. Although there was also a direct correlation with bone metastasis near to the time of surgery, this was because no positive bone scans occurred in patients whose tumours yielded little total PG-LM (less than 16 ng PGE2 equivalents per g tissue). Since tumour PG-LM did not predict later spread to bone, and yields of greater than 16 ng g-1 were similar in the positive and negative bone scan groups, tumour PG-LM appears to be unimportant for skeletal metastasis. There was no obvious relationship of tumour PG-LM to the grade of malignancy, tumour type, amounts of fibrous tissue (and therefore malignant cells), invasion of blood vessels and lymphatics or presence of plasma cells. Multivariate analysis indicates that disease-free survival is longest with an intermediate production of tumour total PG-LM. Of the 82 patients now dead, the cause was attributed to metastatic disease in 69 cases. No relationship of PG-LM to the length of survival was seen with univariate or multivariate analysis. However, when just the post-menopausal patients who died within the first 3 postoperative years were analysed, there was a highly significant inverse correlation between the tumour total PG-LM and the time to death. The reason(s) for these different findings on overall survival compared with just the patients who died are not understood, but the results may indicate that one or more other variables must co-exist with a high tumour PG-LM to hasten death.


*Br. J. Cancer* (1989), **59**, 268–275 © The Macmillan Press Ltd., 1989

### Breast cancer, prostaglandins and patient survival

A. Bennett[1], I.F. Stamford[1], D.A. Berstock[1], F. Dische[2], L. Singh[3] & R.P. A'Hern[4]

[1]*Department of Surgery, King's College School of Medicine and Dentistry, The Rayne Institute, 123 Coldharbour Lane, London SE5 9NU;* [2]*Department of Morbid Anatomy, Dulwich Hospital, London;* [3]*Department of Morbid Anatomy, King's College School of Medicine and Dentistry; and* [4]*Cancer Research Campaign, The Rayne Institute, 123 Coldharbour Lane, London, UK.*

**Summary** Prostaglandins may have both undesirable and desirable effects in malignant disease. Their possible roles in breast cancer were studied by examining the relationships between different variables and the amounts of prostaglandin-like material (PG-LM) extracted from 141 breast carcinomas. Univariate analysis indicates a direct correlation with patient age and menopausal status, with a greater yield from cancers of post- compared with pre-menopausal women. Tumours up to 2 cm diameter yielded more PG-LM than those measuring >2–5 cm. Although there was also a direct correlation with bone metastasis near to the time of surgery, this was because no positive bone scans occurred in patients whose tumours yielded little total PG-LM (<16 ng $PGE_2$ equivalents per g tissue). Since tumour PG-LM did not predict later spread to bone, and yields of >16 ng $g^{-1}$ were similar in the positive and negative bone scan groups, tumour PG-LM appears to be unimportant for skeletal metastasis. There was no obvious relationship of tumour PG-LM to the grade of malignancy, tumour type, amounts of fibrous tissue (and therefore malignant cells), invasion of blood vessels and lymphatics or presence of plasma cells. Multivariate analysis indicates that disease-free survival is longest with an intermediate production of tumour total PG-LM. Of the 82 patients now dead, the cause was attributed to metastatic disease in 69 cases. No relationship of PG-LM to the length of survival was seen with univariate or multivariate analysis. However, when just the post-menopausal patients who died within the first 3 postoperative years were analysed, there was a highly significant inverse correlation between the tumour total PG-LM and the time to death. The reason(s) for these different findings on overall survival compared with just the patients who died are not understood, but the results may indicate that one or more other variables must co-exist with a high tumour PG-LM to hasten death.

Many tumours can produce more prostaglandins (PGs) than the normal tissues in which they arise (Bennett, 1979, 1982, 1988). Similarly, more prostglandin-like material (PG-LM), assayed on rat gastric fundus which is most sensitive to $PGE_2$, was extracted from homogenates of human mammary carcinomas than from benign tumours or macroscopically normal breast tissue (Bennet *et al.*, 1977). Various PGs and related substances extracted from breast carcinomas were identified by Stamford *et al.* (1983) using gas chromatography–mass spectrometry; the products were arachidonate, 12-hydroxy-eicosatetraenoate (12-HETE), thromboxane $B_2$, 15-keto-13,14-dihydro-$TXB_2$, 6-keto-$PGF_{1\alpha}$, 6,15-diketo-$PGF_{1\alpha}$, 615-diketo-13,14-dihydro-$PGF_{1\alpha}$, $PGD_2$, $PGE_2$ and $PGF_{2\alpha}$. Quantitative gas chromatography–mass spectrometry showed substantial amounts of 6-keto-$PGF_{1\alpha}$ and some $PGD_2$, $PGE_2$ and $PGF_{2\alpha}$, with much higher yields of arachidonate (Stamford *et al.*, 1986). It might be expected that these potent substances contribute to breast cancer, but the problem is complex because PGs have numerous actions that may produce both undesirable and desirable effects, and because PGs are formed by both host and malignant cells in the tumour (Bennett, 1979, 1982, 1988).

Several years ago we reported that the production of PG-LM by human primary breast carcinomas was higher in patients with scintigraphic evidence of skeletal metastases and correlated with early death (Bennett *et al.*, 1975, 1977). This study, which closed in 1978 after being extended to 141 patients, confirms the higher median amount of PG-LM from tumours of patients with positive bone scans. However, this is because only negative scans occurred when the tumour PG-LM production was very low. We also confirm the correlation with early death, but we have now found that it does not apply to later death or to premenopausal women. Thus a high yield of PG-LM by the tumours is not necessarily bad, and a low yield is not necessarily good: indeed multivariate analysis indicates that the longest disease-free survival is associated with an intermediate production of tumour total PG-LM.

Correspondence: A. Bennett.
Received 21 September 1988.

### Patients and methods

Samples of histologically confirmed malignant breast tumours were obtained from 152 unselected women undergoing breast surgery in six south-east London hospitals between August 1974 and January 1978. However, 11 were excluded from all analyses: seven had a previous malignancy, three had bilateral breast carcinomas and one patient was irradiated preoperatively. This left a total of 141 patients, but different numbers were available for various analyses, as specified in the relevant sections. The carcinomas were divided into three groups: infiltrating ductal carcinomas ($n=106$), intraduct (non-invasive, $n=4$) and miscellaneous ($n=16$, comprising seven mucinous, four anaplastic, two tubular, one adenocarcinoma with squamous metaplasia, one spheroidal cell carcinoma and one medullary carcinoma). There were four unidentified tumours, and a further 11 cases in which sections were not available.

Independent examinations of the 130 tumours for which sections were available were made by two senior pathologists who were unaware of the PG-LM data. Tumour size, histological type, grade of malignancy, border of growth, lymph node involvement, amount of cellular infiltration around and in the tumour, predominant inflammatory cell type, content of plasma cells, amount of fibrous tissue and the invasion of blood vessels and lymphatics were recorded where possible and scored. The histology was carried out on the part of the tumour retained by the pathologists and is therefore not necessarily representative of the separate portion that was extracted.

All but one of the patients were staged clinically using the TNM classification (stage I=T1–2 N0 M0; stage II=T1–2 N1 M0; stage III=T3–4 N0–2 M0; stage IV=T1–3 N0–2 M1. Forty-five patients (clinical stages I and II) were also staged histologically.

Within 10–60 min of tumour excision, a sample of the malignant tissue provided by a pathologist was homogenised in Krebs solution or acidified ethanol (Bennett *et al.*, 1973, 1977) and extracted for PGs by the method of Unger *et al.* (1971), which gives recoveries of at least 70%. Basal and total PG-LM (total = basal + newly synthesised PG-LM) were bioassayed on rat gastric fundus strips against $PGE_2$ as described in the latter references. Basal values reflect the amount of PG-LM present in the tissues at the time of homogenisation, whereas total PG-LM reflects in addition the new synthesis of PGs from precursors released during homogenisation; the actual amount of the newly synthesised PG-LM is 'total minus basal PG-LM', since apart from the hydrolysis of $PGI_2$ there is little or no degradation of PGs during homogenisation. These values are expressed as ng $PGE_2$ equivalents per g wet weight tissue, and presented as median values with semiquartile ranges in parentheses. In 125 cases the total, basal and synthesised tumour PG-LM were all measured, but in the remaining 16 cases only total PG-LM was determined because of insufficient tissue.

The first skeletal scintigraphy was carried out on 120 patients within 6 months of operation, and on 11 other patients 9–36 months postoperatively, using 5–10 mCi $^{99}$Tc-labelled ethanehydroxy-diphosphonate i.v. Fifteen patients have been excluded from the main analysis because the first scans were either before operation (1 month before in three patients, 5 months before in one patient), or >6 months after surgery (11 patients). The remaining group, scanned up to 6 months postoperatively, has been split into those untreated before the first recurrence and those given various types of therapy (see below). A further 125 scans on 66 of these patients were subsequently carried out at intervals. Before disclosing the PG results, the scans were interpreted by staff of our nuclear medicine department and by surgeons. Recurrences and survival follow-up data were obtained from medical records, the radiotherapy department, general practitioners and the South Thames Cancer Registry.

Various postoperative treatments were given to 67 patients before recurrent disease was detected, and 66 received none; eight have been excluded because the information is not available. The treatment consisted of cytotoxic chemotherapy (usually methotrexate and melphalan), tamoxifen 10 or 20 mg twice daily, non-steroidal anti-inflammatory drugs, oophorectomy, anxiolytic/antidepressant drugs and/or radiotherapy.

Patient survival was measured to the nearest month after surgery (except for the death at 4 days). The times from operation to this analysis are 90–131 months, and the follow-up period extends to 124 months (median 58 months).

*Statistics*

The Mann-Whitney $U$ test, Spearman rank correlation or $\chi^2$ test with Yates' correction (all two-tailed) were used as appropriate. Disease-free interval and patient survival were analysed mainly using the method of Lee & Desu (1972) (SPSS, London University Computer). Multivariate analysis (BMDP, University of California, 1981) with Cox's regression, which assesses the simultaneous influences of several variables, was used to analyse disease recurrence and patient survival. As there are numerous comparisons with total, basal and synthesised PG-LM, for simplicity some are not reported when $P>0.1$.

### Results

Overall, the tumours from the six hospitals yielded similar amounts of PG-LM.

*PG-LM in relation to known prognostic variables*

*Histology* Tumours of different types yielded mainly similar amounts of PG-LM per g wet tissue (Table I), except possibly the four intraduct carcinomas, which tended to produce less.

There was no correlation of grade of malignancy with PG-LM ($n=125$), but the tumour grade correlated inversely with survival (overall comparison $P=0.049$; Lee & Desu, 1972).

Amounts of fibrous tissue (and therefore conversely of malignant tissue), scored as either 0 or + (together forming group 1, $n=54$) or ++(group 2, $n=72$), did not correlate with tumour PG-LM.

**Table I** Amounts of PG-LM in different tumour types

| | Tumour type | | |
|---|---|---|---|
| *PG-LM* | *Infiltrating ductal* | *Intraduct* | *Miscellaneous* |
| Total | 45(16–94) $n=106$ | 22, 4, 29, 13 | 35(13–80) $n=16$ |
| Synthesised | 22(7–54) $n=95$ | 4, 11, 4 | 17(9–35) $n=12$ |
| Basal | 11(3–32) $n=95$ | 0, 18, 9 | 15(4–58) $n=12$ |

Amounts of PG-LM, expressed as ng $PGE_2$ equivalents per g wet weight of tumour, are shown as medians with semiquartile ranges in parentheses or actual values for the intraduct carcinomas. $P>0.1$ for all comparisons between tumour types, except for total PG-LM in infiltrating ductal carcinomas and intraduct tumours where $P=0.09$.

Malignant cells were found in the blood vessels and/or lymphatics of 15/126 tumours. There was no correlation with tumour PG-LM, contrary to the conclusion of the pathologist in our previous paper (Bennett *et al.*, 1977) that of the 55 tumours then available (and included in the present study) 41 showed spread of malignant cells into the blood vessels and/or lymphatics. However, on re-evaluation, the present pathologists found no vessel invasion in 24 formerly considered positive, and they classified three as positive that were previously considered to be negative.

There were 11 tumours with plasma cells and 115 without. Basal tumour PG-LM was higher in the positive group ($P=0.05$) but there was no correlation with the other PG-LM measurements. Analysis of cellular infiltration, predominant inflammatory cell type and border of tumour growth (infiltrating or pushing) in relation to PG-LM were not feasible since in each case one group had very few numbers.

*Tumour size* Tumour diameters were grouped as small, medium and large (up to 2, 2–5 and >5 cm, $n=43$, 50 and 38 respectively). The yield of total PG-LM from small tumours, shown as the median ng $PGE_2$ equivalents $g^{-1}$ with the semiquartile range in parentheses, was 55 (22–95) ng. This was greater than the 22 (10–74) ng $g^{-1}$ from the medium group ($P=0.03$), but mainly similar to the 38 (10–92) ng $g^{-1}$ from large tumours ($P=0.3$).

*Lymph node status* Of the 84 patients examined for malignant lymph nodes, 35 had between 1 and 3, and 11 patients had at least 4. There was no obvious relationship to the primary tumour PG-LM.

*Clinical stage* The numbers of patients in clinical stages I–IV at presentation were respectively 67, 32, 30 and 11 (one not staged). In general, less total PG-LM was obtained from the tumours of stage I patients (stage I versus stages II, III and IV, respectively, $P=0.04$, 0.09 and 0.39; stage I versus the other stages combined $P=0.02$). Their amounts of tumour total PG-LM were respectively 29 (13–79), 59 (27–100), 68 (21–113) and 54 (26–75) ng $g^{-1}$. Similar findings were obtained when just the synthesised PG-LM was analysed, but there was no relationship to basal PG-LM. As expected, stage correlates inversely with the survival time ($P=0.008$).

*Menopausal status* There were 91 post-menopausal patients (last menstrual period at least 2 years before surgery) and 37 premenopausal women. The 13 patients of unclear status have been omitted from the analyses. Tumours from the post-menopausal patients yielded more total PG-LM than those from the premenopausal subjects (44(16–101) versus 19 (9–55) ng $g^{-1}$; $P<0.05$).

*Age* Tumour total PG-LM shows some correlation with the age at the time of surgery in all patients grouped together ($r_s$ 0.19; $P=0.011$, $n=141$), and in the post-menopausal group alone ($r_s$ 0.19; $P=0.034$, $n=91$), but not in premenopausal subjects ($r_s$ 0.11; $P=0.25$, $n=37$).

*Prostaglandins in relation to disease recurrence*

*Bone metastasis near to the time of surgery* The prevalence of positive bone scans near to the time of breast surgery correlates with total and synthesised PG-LM but not with basal PG-LM, regardless of whether treated and untreated patients are assessed separately or together (Table II). The reason for the correlation is that no positive scans occurred when the tumour total PG-LM yield was very low (<16 ng $PGE_2$ equivalents $g^{-1}$). Above this value there was no relation to bone scans, and indeed the highest value was in a scan-negative patient (Figure 1).

Of the 19 patients who were positive when first scanned 0–6 months postoperatively, 15 later presented with symptomatic bone metastases; three patients were negative when rescanned 12–52 months later and did not develop symptomatic bone metastases; the other patient died 2 months postoperatively and her symptomatology is not known.

*Later recurrence in bone* Forty-nine patients had no treatment before disease recurrence, 46 received some form of therapy (see **Patients and methods**) and in 21 cases the information is not available. Of the 12 equivocal scans, 4/6 repeat examinations were positive within 13 months from operation and two were negative within 37 months; the other six were not scanned again. On retesting 48 patients originally with negative scans, 18 became positive 7–64 months after operation, six were considered equivocal and 24 remained negative. The total PG-LM from the tumours of the 18 patients who became scan-positive was lower than from the 19 patients who were initially scan-positive, being 27 (11–95) compared to 73 (27–113) ng $PGE_2$ equivalents $g^{-1}$ ($P$=0.026), but mainly similar to the 40 (12–88) ng $g^{-1}$ for the 88 negative-scan patients ($P$=0.69). Other aspects relating to bone are included in the following section.

**Table II** Breast tumour prostaglandins and bone scan evidence of skeletal metastases

| *Bone scans* | *Total* | *Synthesised* | *Basal* |
|---|---|---|---|
| *Treated and untreated patients* | | | |
| Positive | 73(27–113) $n$=19 | 32(21–86) $n$=17 | 19(6–34) $n$=17 |
| Equivocal | 74(33–120) $n$=9 | 51(27–129) $n$=7 | 18(9–43) $n$=7 |
| Negative | 40(13–88) $n$=88 | 20(6–40) $n$=76 | 12(4–40) $n$=76 |
| *Untreated patients* | | | |
| Positive | 72(19–98) $n$=12 | 34(19–74) $n$=12 | 19(5–20) $n$=12 |
| Equivocal | 74(65–120) $n$=5 | 51(47–108) $n$=5 | 18(12–23) $n$=5 |
| Negative | 36(11–74) $n$=37 | 19(3–40) $n$=32 | 18(4–33) $n$=32 |

These results are for the 116 patients scanned from 0 to 6 months postoperatively. Some received the treatments described in the **Patients and methods** section. Statistical probabilities of $P$>0.1 are not reported. The results from all patients (treated and untreated) were: total PG-LM, positive versus negative $P$=0.026; synthesised PG-LM positive versus negative $P$=0.03; negative versus equivocal $P$<0.03. With just the untreated patients, the statistical probabilities for total PG-LM were: positive versus negative $P$=0.03, equivocal versus negative $P$=0.036; for synthesised PG-LM positive versus negative $P$=0.004; negative versus equivocal $P$=0.015.

**Figure 1** Scintigraphy for bone metastases. Each patient (●) was assessed as positive (+ve), negative (−ve) or equivocal. The different median values of tumour total PG-LM in the +ve and −ve groups ($P$<0.02) occurred because there were no positive scans when the PG-LM was <16 ng $PGE_2$ equivalents $g^{-1}$.

*The incidence of tumour recurrence at any single site* At the time of the last follow-up of all women regardless of menopausal status, there was at least one recurrence in 86 patients (46 local, 57 bone and 21 visceral sites). Tumour total PG-LM in the 48 patients with metastasis at only one site (excluding patients initially stage IV) tended to be highest with tumours that spread locally and lowest with those that spread to the viscera (respectively 78 (9–103) ng $g^{-1}$, $n$=19; and 29 (4–74) ng $g^{-1}$, $n$=8; $P$=0.09). The 21 tumours that metastasised to bone occupied the intermediate position (total PG-LM 51 (23–94) ng $g^{-1}$). As expected, the time to first recurrence correlates with time to death ($r_s$ 0.64; $P$=0.001, $n$=67 excluding the 13 non-cancer deaths and the two cancer deaths where no recurrence was detected). The median recurrence time in bone was shorter (12 months) than locally (26 months, $P$<0.001) or in the viscera (26 months, $P$=0.038). The total, basal or synthesised PG-LM did not correlate with the time to first recurrence (up to 83 months, $P$>0.28) or predict metastasis. However, when only those patients with recurrent disease were analysed, early recurrence (within 18 months) regardless of site tended to correlate inversely with the primary tumour total PG-LM ($P$=0.06). When only the post-menopausal women were examined, this correlation increased ($r_s$ −0.5; $P$=0.016, $n$=20). In contrast, with the small group of premenopausal women there was a weak positive correlation ($r_s$ 0.39; $P$=0.14, $n$=8).

*Disease-free survival* In order to examine further whether there is any relationship of tumour PG-LM to disease-free survival, the time to recurrence (including death without detected recurrence) was analysed further by dividing the patients into groups whose tumour total PG-LM was above or below the median value of 43 ng $g^{-1}$, and into three equal groups of low, intermediate and high total PG-LM (respectively up to 20, 20–80 and >80 ng $PGE_2$ equivalents per g tumour). The patients in the intermediate group have a median disease-free survival of 53.3 months, compared with 27.5 months in the high group ($P$=0.046; Lee & Desu analysis) and 26.5 months in the low group ($P$=0.11). This finding is mainly similar to the multivariate analysis below (disease-free interval section).

*Patient survival*

*All patients* Of the 82 deaths (58 post-menopausal, 17 premenopausal and seven of unclear status), 69 were reported as due to cancer and 13 were attributed to other causes. Survival curves (data from both dead and alive patients) were similar when the patients were grouped above or below the median tumour total PG-LM value, or into low, intermediate or high total tumour PG–LM groups as above (Figure 2). Tumour PG-LM values of the 63 patients who survived 6 years or more are distributed similarly above and below the median value for all patients.

However, on analysing just the patients dying within 3 years of surgery (a time point suggested by the data and used here for hypothesis generation), there is a highly significant inverse correlation with tumour total PG-LM for the 39 dying from cancer ($r_s$ −0.55) and for the 46 deaths from all causes ($r_s$ −0.49) (both $P<0.001$; Figure 3). The relationship does not hold after the 3-year period.

*Premenopausal women* The median survival time was 31 months. There was a weak trend for a positive correlation between total PG–LM and time to death (all reportedly from cancer) in the 17 premenopausal patients who have now died ($P=0.11$), and in the 10 patients dying within 3 years ($P=0.21$; Figure 4a). Survival curves (i.e. including alive and dead patients) were similar in patients with total, basal or synthesized PG-LM values above and below their respective median values, or divided into low, medium and high groups, except that survival tended to be longest with the highest total PG-LM ($P=0.09$).

*Post-menopausal patients* As with the premenopausal women, the median survival time was 31 months. However, unlike this group, the time to death up to 3 years post-operatively showed striking inverse correlations with total, basal and synthesised tumour PG-LM (respectively $r_s$ −0.6; $P=0.001$, $n=34$; Figure 4b; $r_s$ −0.51; $P=0.002$, $n=30$; $r_s$ −0.42; $P=0.01$, $n=30$). After the 3-year period, there was no relationship between the total PG-LM and time to death ($r_s$ −0.049; $P=0.36$, $n=56$).

Because of the striking relationship of PG-LM to early death, we examined several variables for possible differences between the 35/91 (38%) of post-menopausal women dying within 3 years compared with the 62% surviving longer (Table III). Of those living longer, more had stage I tumours and less were stage IV; more had grade 1 tumours; recurrence occurred later with more frequent local spread and less in bone. There was little or no difference in the initial bone scan finding, tumour PG-LM, age at the time of surgery, lymph node involvement, tumour size, additional treatment, histological type or amounts of fibrous tissue (Table III).

*Multivariate analysis of factors that may affect disease recurrence and patient survival*

Only those variables of proven prognostic value were included (age, bone scan result and stage), to avoid the problem of multiple significance testing and because of some missing data. Menopausal status was not added as it contributes to the included variable of age. Tumour size and nodal status were not included partly because they contribute to stage and partly because the results were not available for all patients. Tumour grade was omitted because of its low contribution to prognosis in the presence of the other variables analysed. Pathological staging was used in preference to clinical staging whenever possible, since it is more accurate. Table IV shows the variables included in the analysis; none of them interacted. The values given below are followed by the 95% confidence limits in parentheses.

*Disease-free interval* In general agreement with the Lee & Desu analysis, the intermediate group experienced 50% (28–

**Figure 2** Patient survival was similar in the groups with low (▮▮▮▮), medium (////) or high (▬▬) amounts of tumour total PG-LM (respectively up to 20, 20–80 and >80 ng $PGE_2$ equivalents per g tumour).

**Figure 3** The time to death of all patients (cancer death ●, $n=69$; non-cancer death ○, $n=13$) is inversely related to the tumour total PG-LM up to 3 years postoperatively ($P<0.001$), but not subsequently.

**Figure 4** The time to death of premenopausal patients up to 3 years postoperatively shows, if anything, a weak correlation with tumour total PG-LM ($P=0.21$; **a**). In contrast, the post-menopausal patient deaths up to 3 years postoperatively are inversely related to the tumour total PG-LM ($P<0.001$; **b**).

90% confidence limits) of the hazard in the low total PG-LM group ($P<0.05$), and 55% (31–98%) of that in the high group ($P<0.05$). There was no relationship of PG-LM to just the bone scan data.

**Table III** Variables in post-menopausal patients surviving for up to 3 years after breast surgery compared with those living longer

| | *Survival up to 3 years* | *Survival >3 years* | *P value (Mann–Whitney or $\chi^2$)* |
|---|---|---|---|
| Number of patients | 35/91 (38%) | 56/91 (62%) | |
| *Tumour PG-LM (ng $PGE_2$ equiv. $g^{-1}$)* | | | |
| Total | 40(16–88) $n=35$ | 48(16–94) $n=56$ | 0.92 (MW) |
| Basal | 8( 3–24) $n=31$ | 12( 4–29) $n=47$ | 0.49 (MW) |
| Synthesised | 22( 7–54) $n=31$ | 28( 9–65) $n=47$ | 0.45 (MW) |
| *Tumour stage* | | | |
| I | 9/35 (26%) | 33/55 (60%)[a] | 0.0019 ($\chi^2$) |
| II | 8/35 (23%) | 7/55 (13%) | |
| III | 11/35 (31%) | 14/55 (25%) | |
| IV | 7/35 (20%) | 1/55 ( 2%) | |
| *Tumour grade* | | | |
| 1 | 6/34 (18%) | 21/49 (43%) | 0.052 ($\chi^2$) |
| 2 | 22/34 (64%) | 21/49 (43%) | |
| 3 | 6/34 (18%) | 7/49 (14%) | |
| *Tumour size* | | | |
| <2 cm | 9/30 (30%) | 21/40 (53%) | 0.15 ($\chi^2$) |
| 2–5 cm | 17/30 (57%) | 14/40 (35%) | |
| >5 cm | 4/30 (13%) | 5/40 (12%) | |
| *Histol. type* | | | |
| Infiltrating | 28/33 (85%) | 41/50 (82%) | 0.85 ($\chi^2$) |
| Intraduct | 1/33 ( 3%) | 1/50 ( 2%) | |
| Others | 4/33 (12%) | 8/50 (16%) | |
| *Fibrous tissue* | | | |
| 0 or + | 16/33 (48%) | 22/50 (44%) | 0.86 ($\chi^2$) |
| ++ | 17/33 (52%) | 28/50 (56%) | |
| *Positive lymph nodes* | | | |
| 0 | 7/24 (29%) | 16/30 (53%) | 0.18 ($\chi^2$) |
| 1–3 | 12/24 (50%) | 11/30 (37%) | |
| 4+ | 5/24 (21%) | 3/30 (10%) | |
| Recurrence (months)[b] | 11(9–15) $n=20$ | 39(26–51) $n=22$ | <0.0001 (MW) |
| *Spread*[b] | | | |
| Local | 7/29 (24%) | 15/26 (58%) | 0.02 ($\chi^2$) |
| Visceral | 18/29 (62%) | 7/26 (27%) | |
| Bone | 4/29 (14%) | 4/26 (15%) | |
| *Spread to only one site*[c] | | | |
| Local | 3/15 (20%) | 10/16 (62%) | <0.05 ($\chi^2$) |
| Visceral | 4/15 (27%) | 3/16 (19%) | |
| Bone | 8/15 (53%) | 3/16 (19%) | |
| *First bone scan* | | | |
| Negative | 18/30 (60%) | 41/52 (79%) | 0.096 ($\chi^2$) |
| Equivocal | 6/30 (20%) | 3/52 ( 6%) | |
| Positive | 6/30 (20%) | 8/52 (15%) | |
| Age at surgery (years) | 68(59–74) $n=35$ | 65(57–74) $n=56$ | 0.58 (MW) |
| *Non-surgical postoperative therapy* | | | |
| Drugs | 3/35 ( 8%) | 9/52 (17%) | 0.12 ($\chi^2$) |
| Radiotherapy | 10/35 (29%) | 12/52 (23%) | |
| Radiotherapy + drugs | 6/35 (17%) | 2/52 ( 4%) | |
| None | 16/35 (46%) | 29/52 (56%) | |

Compared with the patients living up to 3 years postoperatively, the longer surviving patients more often had tumours of a lower stage or grade and a later recurrence. There was little or no relationship to the histological type, amount of fibrous tissue (and therefore conversely of malignant tissue), initial bone scan finding, tumour PG-LM, age at surgery, lymph node status, tumour size or non-surgical treatment.

[a]One patient not staged; [b]regardless of the number of recurrence sites per patient; [c]patients with recurrence at only one site, excluding stage IV.

*Survival* The group with intermediate total PG-LM showed at most a weak tendency to survive longer than the low and high PG-LM groups. The hazard for the intermediate group was 66 (38–115)% of that for the low group ($P=0.16$), and 79 (44–143)% of that for the high group ($P=0.44$).

### Discussion

We have confirmed that human mammary carcinomas can usually produce substantial amounts of prostaglandin-like material (PG-LM), although we do not know the relative

**Table IV** The probability values associated with the prognostic variables in the multivariate analysis of disease recurrence and survival

| *Variable* | *Recurrence* | *Survival* |
|---|---|---|
| Bone scan | 0.001[a] | 0.24 |
| Stage | 0.012[b] | 0.026 |
| Total PG-LM | 0.035 | 0.033 |
| Age | 0.77 | 0.36 |

All the factors shown were included in the regression model (see Gore *et al.*, 1984) because of their possible importance as prognostic indicators. The analysis of disease-free survival excludes patients with positive bone scans and stage IV disease.

[a]Equivocal versus negative; [b]excluding stage IV disease.

contributions from malignant and host cells. Many factors hamper the interpretation of our results, including the problems of measuring and identifying the source of tumour PG-LM. The measurement problem would be less with the current better methods, but only bioassay was available to us when our study began, and various important prostanoids such as $PGI_2$ and thromboxane $A_2$ had not yet been discovered. Our bioassay is most sensitive to $PGE_2$ (Bennett *et al.*, 1980), and the measurements of tumour PG-LM probably reflect mostly this compound. However, breast tumours can produce several different PGs and related substances (Stamford *et al.*, 1983). Indeed, the amount of $PGF_{2\alpha}$, to which bioassay is about 10 times less sensitive (Bennett *et al.*, 1980), was similar to that of $PGE_2$ (Fulton *et al.*, 1982; Watson *et al.*, 1984).

A simple relationship between cancer and PGs is unlikely because these numerous substances can have diverse (sometimes even opposite) effects on cell proliferation, differentiation, host defence and metastasis, etc. (Bennett, 1979, 1982, 1989). Furthermore, some PGs are involved in inflammation, which has a variable effect on tumour spread, a small inflammatory reaction causing enhancement and a large one causing inhibition (Van den Brenk *et al.*, 1974).

*Prostaglandins in relation to known prognostic variables*

Univariate analysis shows that tumour PG-LM correlates directly with patient age, menopausal status and incidence of positive bone scintiscans near to the time of surgery, but inversely with tumour size. The bone aspects were an important reason for starting the study, and are dealt with first.

*Bone metastasis* We previously reported that breast cancers of patients whose isotopic bone scans were positive near to the time of surgery yielded a higher median amount of total PG-LM than the negative group (Bennett *et al.*, 1977). The same is true in the present larger study, which incorporates the previous results, but the correlation occurs only because there were no positive initial bone scans in patients whose tumours yielded very low amounts of PG-LM ($<16\,\mathrm{ng\,g^{-1}}$, Figure 1). Above this value the tumour PG-LM values are similar in the positive and negative groups. Furthermore, the tumour PG-LM was not related to subsequent metastasis to bone. Thus tumour PGs seem to be unimportant in the spread of tumour to bone.

Even though breast carcinomas may release PGs into the blood (Stamford *et al.*, 1980), the concentrations are probably insufficient to resorb bone, particularly since any PGE and F compounds would be mainly inactivated during passage through the pulmonary circulation (Ferreira & Vane, 1967). The tumours may also release $PGI_2$ (Demers *et al.*, 1979), another potent bone resorber (Ali *et al.*, 1979), but although $PGI_2$ survives the pulmonary circulation (Dusting *et al.*, 1978) and is sufficiently stable for some to reach the skeleton, amounts needed to affect bone would presumably exceed those causing profound hypotension. Thus if PGs have any role as resorbing agents in bone metastasis, it is probably only those formed locally by the metastasised cells that are involved. Our multivariate analysis, and studies with PG synthesis inhibitors, also argue against a role for PGs in bone metastasis. Although indomethacin can reduce some other malignant hypercalcaemias (Seyberth *et al.*, 1975), it did not affect hypercalcaemia in breast cancer (Coombes *et al.*, 1976), and benorylate did not affect the skeletal spread of breast cancer (Powles *et al.*, 1980). However, we must take care before coming to a firm conclusion. PGs *in vivo* can, unlike *in vitro*, cause bone formation (Ueda *et al.*, 1980), and cyclo-oxygenase inhibitors may increase the metabolism of PG precursors into lipoxygenase products (Higgs *et al.*, 1980), some of which potently resorb bone *in vitro* (Meghji *et al.*, 1988). Furthermore, skeletal scans are not always reliable indicators of bone metastases (Coombes *et al.*, 1983), and this might explain why a few of our scan-positive patients were negative on subsequent retesting.

*Menopausal status* Cancers from post-menopausal women yielded more PG-LM compared with the premenopausal group. This agrees with Fulton *et al.* (1982) who obtained more $PGE_2$ from tumours of post-menopausal women compared with those who were pre- or perimenopausal. However, Watson *et al.* (1984) found similar yields of both $PGE_2$ and $PGF_{2\alpha}$.

*Age* Overall, the breast tumour total PG-LM correlates with age. Similar trends were seen for microsomal $PGE_2$ formation (Rolland *et al.*, 1980) or for $PGF_{2\alpha}$ production (Vergote *et al.*, 1985), but Kibbey *et al.* (1979) found no relationship to $PGE_2$. Fulton *et al.* (1982) obtained no correlation of $PGE_2$ with age in just the post-menopausal women, whereas we did so with PG-LM in this group but not in premenopausal subjects.

*Tumour size* Overall, the tumour size correlated inversely with PG-LM production, in agreement with Rolland *et al.* (1980) who measured microsomal $PGE_2$ formation from added arachidonic acid, with Fulton *et al.* (1982) who measured endogenously formed $PGE_2$, and with Vergote *et al.* (1985) who found a similar tendency with $PGF_{2\alpha}$. In contrast, Karmali *et al.* (1983) and Watson *et al.* (1987) found no relationship between PGs and tumour size. Our inverse correlation with PG-LM was due mainly to the results with only the small and medium tumours. Although ischaemia and necrosis of the central part of larger tumours might affect the formation of PGs, we washed away the necrotic tissue before extraction.

*Variables showing no relationship to PG-LM* It must be stressed that the samples for histology or extraction were from different parts of the tumour, and this might explain lack of correlation. Grade of malignancy, the main histological variable that influences prognosis (Bloom & Richardson, 1957), did not correlate with PG-LM. Fulton *et al.* (1982) found that $PGE_2$ and $PGF_{2\alpha}$ correlated with the grade of malignancy, but in contrast Vergote *et al.* (1985) obtained more $PGF_{2\alpha}$ from differentiated tumours than from undifferentiated tumours.

We previously reported a correlation between tumour PG-LM and the histological presence of malignant cells in the blood vessels, lymphatics and lymph nodes (Bennett *et al.*, 1977). Furthermore, Rolland *et al.* (1980) concluded that tumours whose microsomes produced highest amounts of $PGE_2$ from added arachidonic acid were most malignant. Unfortunately, our new histological assessment differs from the previous one, and argues against a relationship between PGs and tumour spread into vessels. Since pathologists vary in their histological assessment of tumour grade (Delides *et*

*al.*, 1982) and malignant cells in blood vessels (4.7–52%; Fisher *et al.*, 1975; Borah *et al.*, 1980; Weigand *et al.*, 1982), this argument is not settled. Furthermore, perhaps a tumour total PG-LM production of at least $16 \mathrm{ng\,g^{-1}}$ aids the dispersion of malignant cells, as judged by the absence of positive bone scans near to the time of surgery in patients whose primary tumours produced very low amounts of total PG-LM.

The tumour type seems to bear at most a weak relationship to the PG-LM yield. However, the four intraduct (non-invasive) carcinomas produced comparatively little PG-LM, consistent with the low contents of $PGF_{2\alpha}$ in comedo (intraduct) tumours (Vergote *et al.*, 1985).

Amounts of fibrous tissue (and therefore conversely of malignant cells), or the presence of plasma cells, did not correlate with PG-LM. With other variables there were insufficient numbers in one group to evaluate the relationship of PG-LM to cellular infiltration, the predominant inflammatory cell type, or border of tumour growth.

We did not measure steroid receptors in our present experiments, but our separate study (Wilson *et al.*, 1980) indicates that the oestrogen receptor content and breast tumour PG-LM are independent variables. Watson *et al.* (1984, 1987) obtained no correlation between $PGE_2$ or $PGF_{2\alpha}$ and oestrogen or progesterone receptors, but Campbell *et al.* (1982) found that oestrogen receptor-positive cancers produced more $PGE_2$ than the receptor-negative tumours, and Vergote *et al.* (1985) found a correlation between $PGF_{2\alpha}$ and both the oestrogen and progesterone status. Karmali *et al.* (1983) reported that tumours with progesterone receptors tended to produce more $PGI_2$ than did receptor-negative tumours; no relationship to other PGs was found, but thromboxane production was lower when oestrogen receptors were present. Fulton *et al.* (1982) found no relationship of tumour $PGE_2$ or $PGF_{2\alpha}$ to progesterone receptors but there was a variable association with oestrogen receptors.

*Disease-free survival*

Tumour PG-LM tends to correlate inversely with recurrence at any site up to 18 months postoperatively ($P = 0.06$), but not subsequently. This is consistent with an absence of positive bone scans with tumours yielding low amounts of total PG-LM. However, multivariate analysis indicates that the longest disease-free interval occurs with an intermediate tumour total PG-LM. Thus a high production of tumour PG-LM is not necessarily bad. Vergote *et al.* (1985) found that a good prognosis was associated with a high yield of tumour $PGF_{2\alpha}$, but the biological effects of this PG are often different, and sometimes opposite, to those of $PGE_2$. In contrast, Watson *et al.* (1987) found no relationship of $PGE_2$ and $PGF_{2\alpha}$ to the disease-free interval.

*Survival*

When 25 patients had died and the follow-up time was 1.5–54 months, we reported an inverse correlation between total PG-LM and the time to death (Bennett *et al.*, 1979). Similarly in the present study, which includes the patients in the previous analysis, there is a highly significant inverse correlation of tumour total PG-LM with the time to death within the first 3 postoperative years. However, we have now found that this correlation occurs only in the post-menopausal women.

The difference between the findings with just the dead patients, compared with the overall survival which includes the alive and dead patients, is a puzzle. Perhaps a high tumour PG-LM leads to early death only when associated with undetermined factor(s), one of which might be oestrogen receptors whose absence carries a poor prognosis (Howat *et al.*, 1985); prognosis might be best with oestrogen receptor-positive tumours that produce an intermediate amount of tumour PG-LM. The 3-year cut-off point is one suggested by the data, since there is no relationship of PG-LM to survival after this time. This study has therefore generated the hypothesis that PG-LM may be a factor in early death from breast cancer. Other studies that are already in progress will be able to test this possibility.

In summary, our main conclusions are that breast tumour PG-LM production (which probably represents mostly $PGE_2$) seems unlikely to be important in metastasis to bone or other sites, or to overall patient survival. Nevertheless, a striking finding is that post-menopausal women dying within 3 years of surgery show a highly significant inverse correlation between tumour total PG-LM and time to death. The reason for this is not understood but, if it is not an artefact, it presumably requires the presence of one or more other variables for the early prognosis to be bad. It is also interesting that no positive bone scans were found near to the time of surgery in patients whose tumours produced low amounts of PG-LM. However, a high production of PG-LM by the tumour is not necessarily bad, and the disease-free survival is longest in women whose breast tumours produced intermediate amounts of total PG-LM.

We thank the CRC, MRC, the Wellcome Trust, King's Medical Research Trust and the Association for International Cancer Research for support, and Dr K. MacRae, Mrs P.B. Melhuish, Dr D. Cooper and numerous other people for their help.

### References

ALI, N.N., AUGER, D.W., BENNETT, A., EDWARDS, D.A. & HARRIS, M. (1979). Effect of prostacyclin and its breakdown product 6-oxo-$PGF_{1\alpha}$ on bone resorption *in vitro*. In *Prostacyclin*, Vane, J.R. & Bergstom, S. (eds) p. 179. Raven Press: New York.

BENNETT, A. (1979). Prostaglandins and cancer. In *Practical Applications of Prostaglandins and Their Synthesis Inhibitors*, Karim, S.M.M. (ed) p. 149. MTP Press: Lancaster.

BENNETT, A. (1982). Prostaglandins and inhibitors of their synthesis in cancer growth and spread. In *Endocrinology of Cancer, Vol. 3*, Rose, D.P. (ed) p. 113. CRC Press: Boca Raton.

BENNET, A. (1989). Prostaglandins and cancer. In *CRC Handbook of Eicosanoids and Related Lipids*, Willis, A.L., Pace-Asciak, C., Vickery, B. (eds). CRC Press: Boca Raton.

BENNETT, A., BERSTOCK, D.A., RAJA, B. & STAMFORD, I.F. (1979). Survival time after surgery is inversely related to the amounts of prostaglandins extracted from human breast tumours. *Br. J. Pharmacol.*, **66**, 451.

BENNETT, A., CHARLIER, E.M., MCDONALD, A.M., SIMPSON, J.S., STAMFORD, I.F. & ZEBRO, T. (1977). Prostaglandins and breast cancer. *Lancet*, **ii**, 624.

BENNETT, A., JAROSIK, C., SANGER, G.J. & WILSON, D.E. (1980). Antagonsim of prostanoid-induced contractions of rat gastric fundus muscle by SC-19220, sodium meclofenamate, indomethacin or trimethoquinol. *Br. J. Pharmacol.*, **71**, 169.

BENNETT, A., MCDONALD, A.M., SIMPSON, J.S. & STAMFORD, I.F. (1975). Breast cancer, prostaglandins and bone metastases. *Lancet*, **i**, 1218.

BENNETT, A., STAMFORD, I.F. & UNGER, W.G. (1973). Prostaglandin $E_2$ and gastric acid secretion in man. *J. Physiol.*, **229**, 349.

BLOOM, H.J.G. & RICHARDSON, W.W. (1957). Histological grading and prognosis in breast cancer: A study of 1,409 cases of which 359 have been followed for 15 years. *Br. J. Cancer*, **11**, 359.

BORAH, V., SHAH, P.N., GOSH, S.N., SAMPAT, M.B. & JUSSAWALLA, D.J. (1980). Further studies on the prognostic importance of Barr body frequency in human breast cancer: With discussion on its probable mechanism. *J. Surg. Oncol.*, **13**, 1.

BMDP STATISTICAL SOFTWARE (1981). Program 2L. Department of Biomathematics, University of California, USA.

CAMPBELL, F.C., HAYNES, J., EVANS, D.F. & 4 others (1982). Prostaglandin $E_2$ synthesis by tumour epithelial cells and oestrogen receptor status of primary breast cancer. *Langenbecks Arch. Chir.*, **357**, 209.

COOMBES, R.C., DADY, P., PARSONS, C. & 4 others. (1983). Assessment of response of bone metastases to systemic treatment in patients with breast cancer. *Cancer*, **53**, 610.

COOMBES, R.C., NEVILLE, A.M. & BONDY, P.K. (1976). Failure of indomethacin to reduce hydroxyproline excretion or hypercalcaemia in patients with breast cancer. *Prostaglandins*, **12**, 1027.

DELIDES, G.S., GARAS, G., GEORGOULI, G. & 4 others (1982). Intralaboratory variations in the grading of breast carcinoma. *Arch. Pathol. Lab. Med.*, **106**, 126.

DEMERS, L.M., SCHWEITZER, J. & LIPTON, A. (1979). Blood 6-keto-$F_{1\alpha}$ levels as potential tumour marker. Poster presentation to the First International Congress on Hormones and Cancer, Rome.

DUSTING, G.J., MONCADA, S. & VANE, J.R. (1978). Disappearance of prostacyclin ($PGI_2$) in the circulation of the dog. *Br. J. Pharmacol.*, **62**, 414.

FERREIRA, S.H. & VANE, J.R. (1967). Prostaglandins, their disappearance from and release into the circulation. *Nature*, **216**, 868.

FISHER, E.R., GREGORIO, R. & FISHER, B. (1975). The pathology of invasive breast cancer. *Cancer*, **36**, 1.

FULTON, A., ROI, L., HOWARD, L., RUSSO, J., BROOKS, S. & BRENNAN, M.J. (1982). Tumour-associated prostaglandins in patients with primary breast cancer: Relationship to clinical parameters. *Breast Cancer Res. Treat.*, **2**, 331.

GORE, S.M., POCOCK, S.J. & KERR, G.R. (1984). Regression models and non-proportional hazards in the analysis of breast cancer survival. *Appl. Stat.*, **33**, 176.

HIGGS, G.A., MUGRIDGE, K.E., MONCADA, S. & VANE, J.R. (1980). Effects of non-steroid anti-inflammatory drugs on leukocyte migration in carrageenin-induced inflammation. *Eur. J. Pharmacol.*, **6**, 81.

HOWAT, J.M.T., HARRIS, M., SWINDELL, R. & BARNES, D.M. (1985). The effect of oestrogen and progesterone receptors on recurrence and survival in patients with carcinoma of the breast. *Br. J. Cancer*, **51**, 263.

KARMALI, R.A., WELT, S., THALER, H.T. & LEFEVRE, F. (1983). Prostaglandins in breast cancer: Relationship to disease stage and hormone status. *Br. J. Cancer*, **48**, 689.

KIBBEY, W.E., BRONN, D.G. & MINTON, J.P. (1979). Prostaglandin synthetase and prostaglandin $E_2$ levels in human breast carcinoma. *Prostaglandins Med.*, **2**, 133.

LEE, E. & DESU, M. (1972). A computer program for comparing K samples with right-censored data. *Computer Prog. Biomed.*, **2**, 315.

MEGHJI, S., SCUTT, A. & HARVEY, W. (1988). Stimulation of bone resorption by lipoxygenase products *in vitro*. *Prostaglandins*, **36**, 139.

POWLES, T.J., DADY, P.J., WILLIAMS, J., EASTY, G.C. & COOMBES, R.C. (1980). Use of inhibitors of prostaglandin synthesis in patients with breast cancer. *Adv. Prostaglandin Thromboxane Res.*, **6**, 511.

ROLLAND, P.H., MARTIN, P.M., JACQUEMIER, J., ROLLAND, A.M. & TOGA, M. (1980). Prostaglandin in human breast cancer: Evidence suggesting that an elevated prostaglandin production is a marker of high metastatic potential for neoplastic cells. *J. Natl Cancer Inst.*, **64**, 1061.

SEYBERTH, H.W., SEGRE, G.V., MORGAN, J.L., SWEETMAN, B.J., POTTS, J.T. & OATES, J.A. (1975). Prostaglandins as mediators of hypercalcaemia associated with certain types of cancer. *N. Engl. J. Med.*, **293**, 1278.

STAMFORD, I.F., BENNETT, A., CIVIER, A. & HENSBY, C.N. (1986). Human cancers yield elevated amounts of arachidonate. 6th International Conference on Prostaglandins and Related Compounds. Florence, Italy. Abstract.

STAMFORD, I.F., CARROLL, M.A., CIVIER, A., HENSBY, C.N. & BENNETT, A. (1983). Identification of arachidonate metabolites in normal, benign and malignant human mammary tissues. *J. Pharm. Pharmacol.*, **35**, 48.

STAMFORD, I.F., McINTYRE, J. & BENNETT, A. (1980). Human breast carcinomas release prostaglandin-like material into the blood. *Adv. Prostaglandin Thromboxane Res.*, **6**, 571.

UEDA, K., SAITO, A., NAKANO, H. & 4 others (1980). Cortical hyperostosis following long-term administration of prostaglandin $E_1$ in infants with cyanotic congenital heart disease. *J. Pediatr.*, **97**, 831.

UNGER, W.G., STAMFORD, I.F. & BENNETT, A. (1971). Extraction of prostaglandins from human blood. *Nature*, **233**, 336.

VAN DEN BRENK, H.A.S., STONE, M., KELLY, H., ORTON, C. & SHARPINGTON, C. (1974). Promotion of growth of tumour cells in acutely inflamed tissues. *Br. J. Cancer*, **30**, 246.

VERGOTE, I.B., LAEKEMAN, G.M., KEERSMAEKERS, G.H. & 6 others (1985). Prostaglandin $F_{2\alpha}$ in benign and malignant breast tumours. *Br. J. Cancer*, **51**, 827.

WATSON, D.M.A., KELLY, R.W., HAWKINS, R.A. & MILLER, W.R. (1984). Prostaglandins in human mammary cancer. *Br. J. Cancer*, **49**, 459.

WATSON, D.M.A., KELLY, R.W. & MILLER, W.R. (1987). Prostaglandins and prognosis in human breast cancer. *Br. J. Cancer*, **56**, 367.

WEIGAND, R.A., ISENBERG, W.M., RUSSO, J., BRENNAN, M.J. & RICH, M.A. (1982). Blood vessel invasion and axillary lymph node involvement as prognostic indicators for human breast cancer. *Cancer*, **50**, 962.

WILSON, A.J., BAUM, M., BENNETT, A., GRIFFITHS, K., NICHOLSON, R.I. & STAMFORD, I.F. (1980). Lymph node status, prostaglandins and oestrogen receptors are independent prognostic variables in human primary breast cancer. *Clin. Oncol.*, **6**, 379.


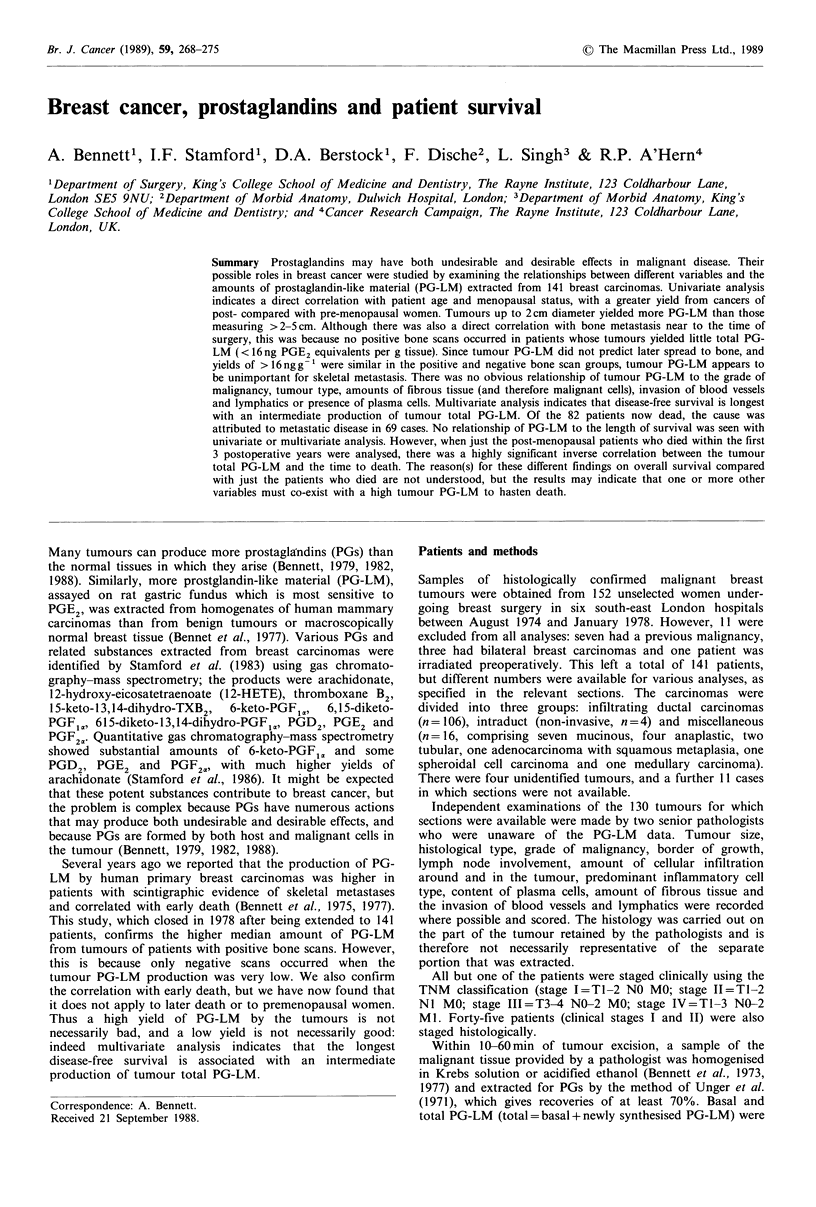

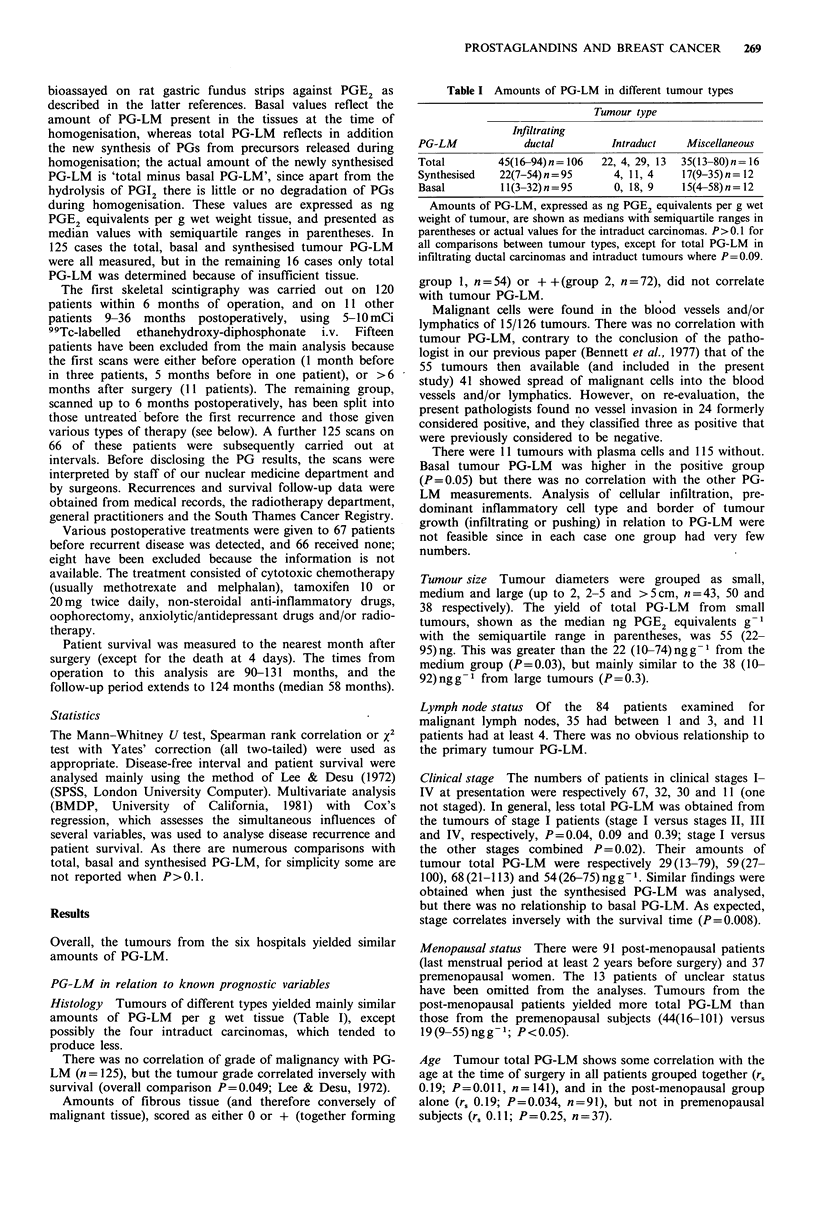

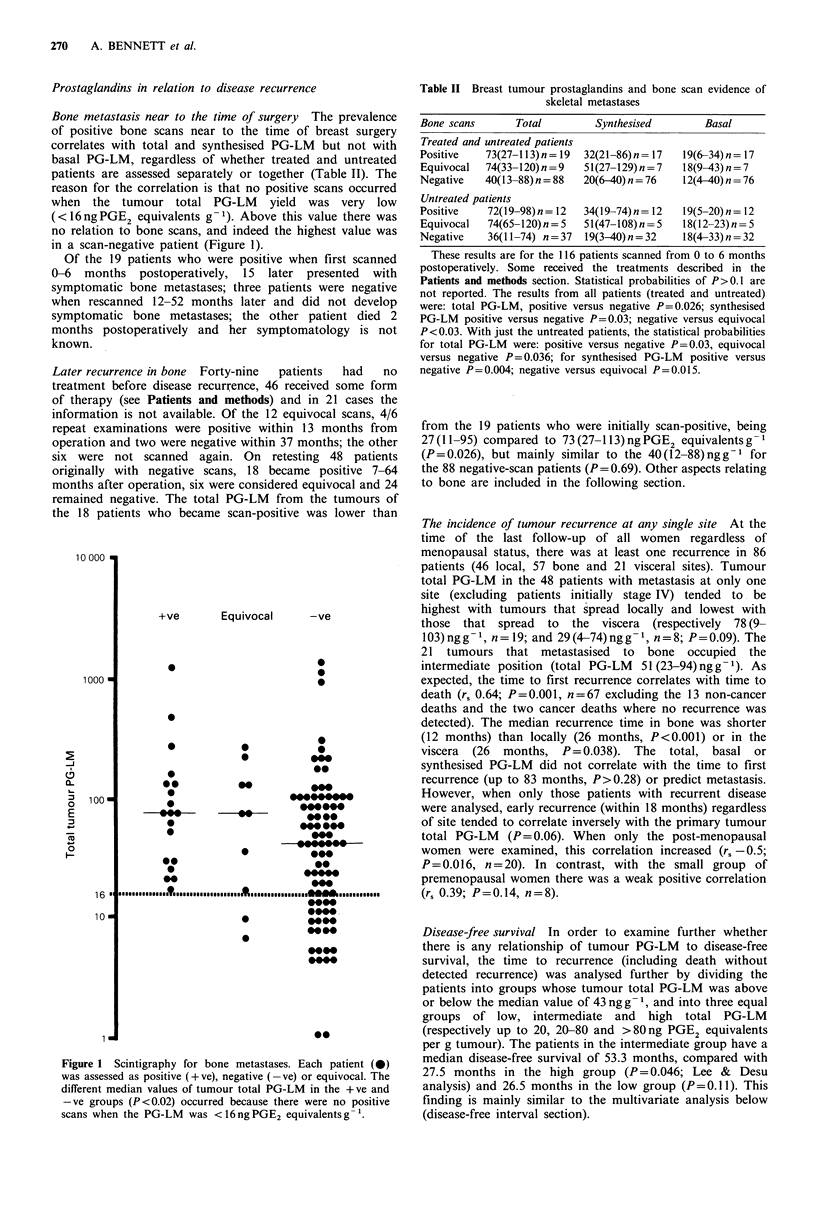

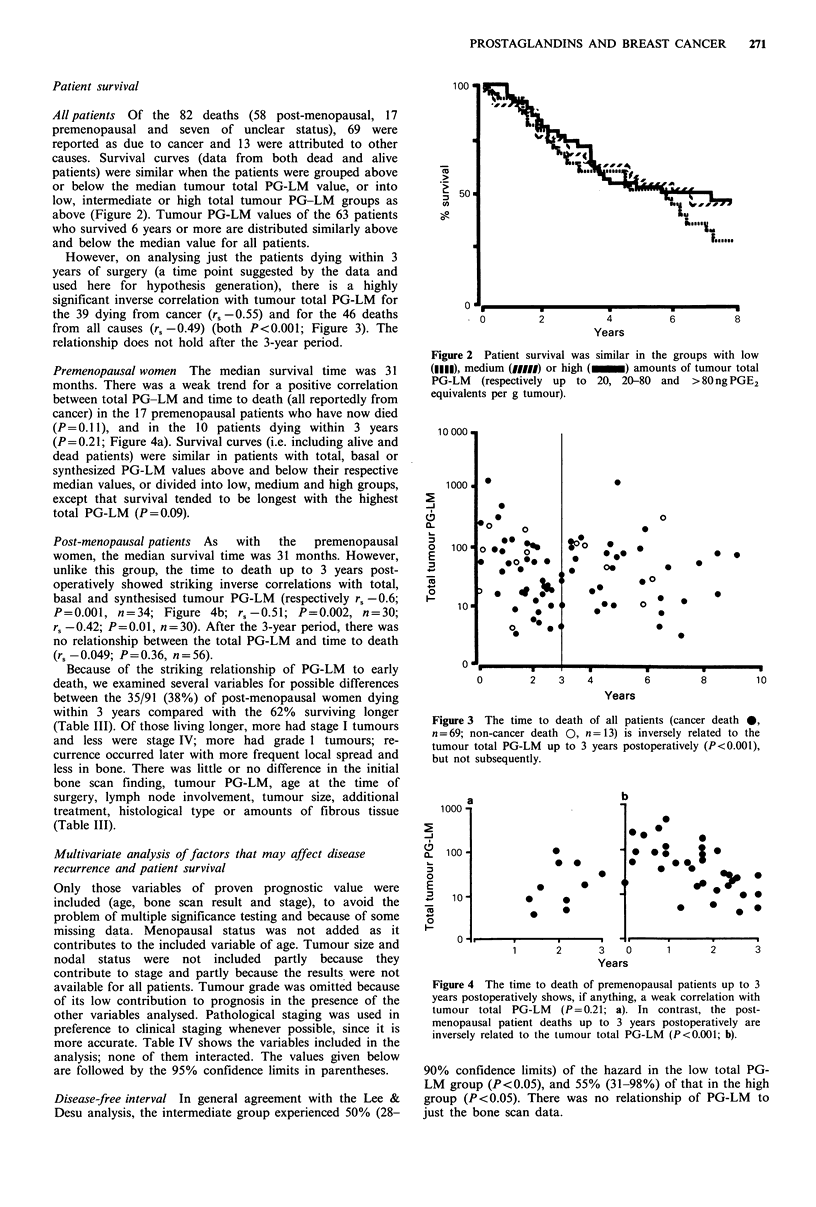

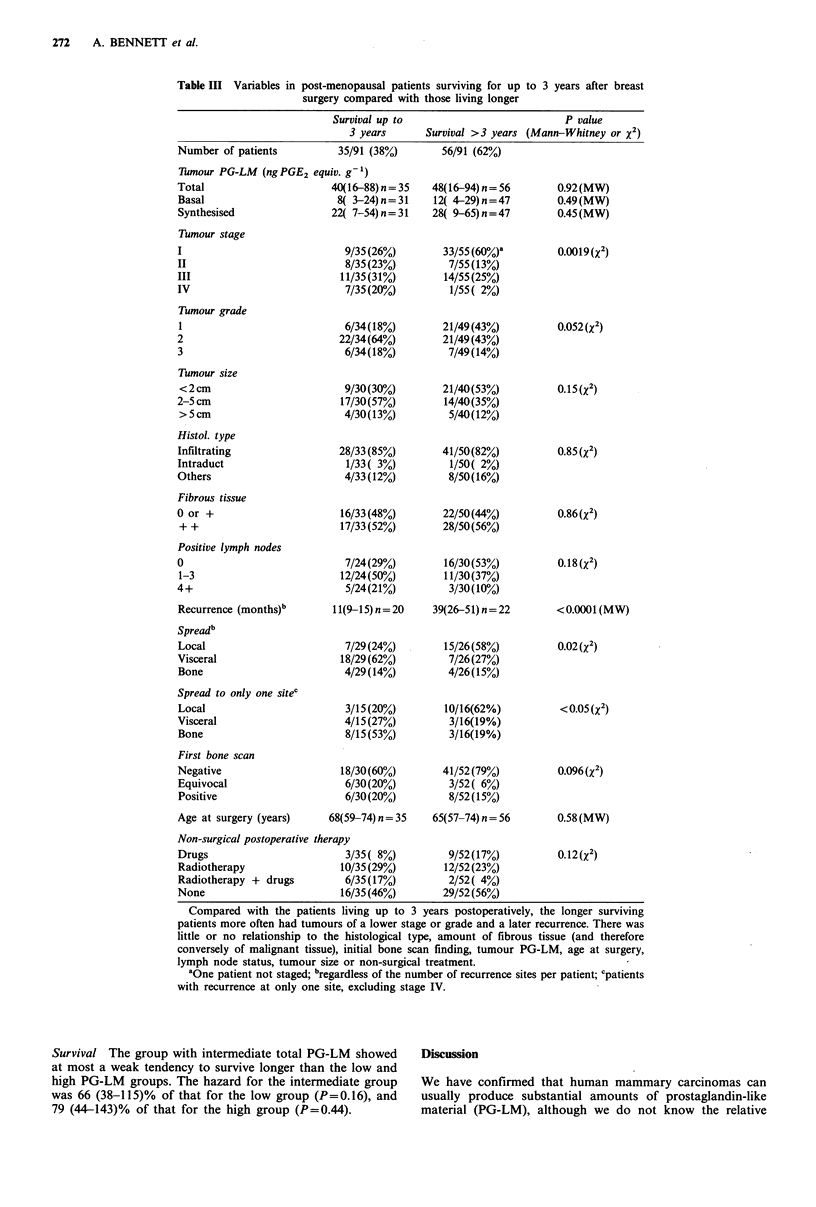

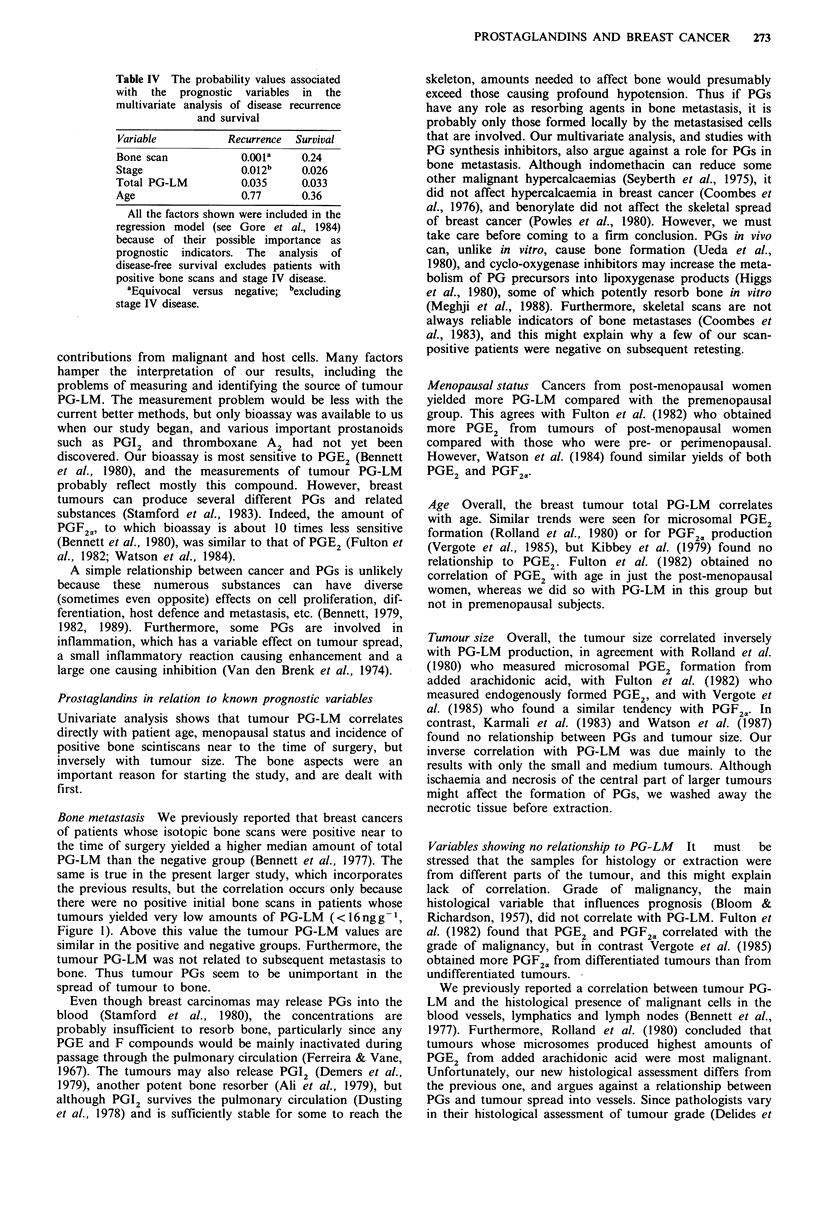

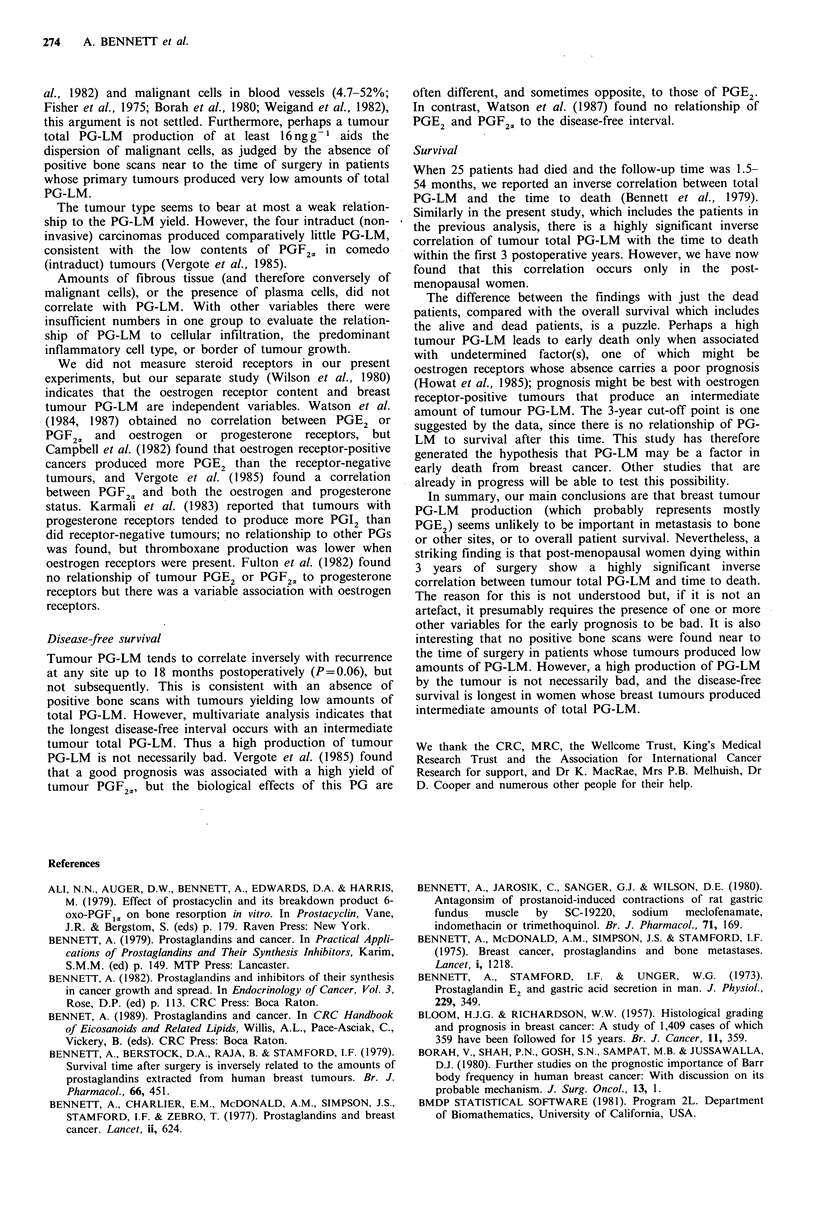

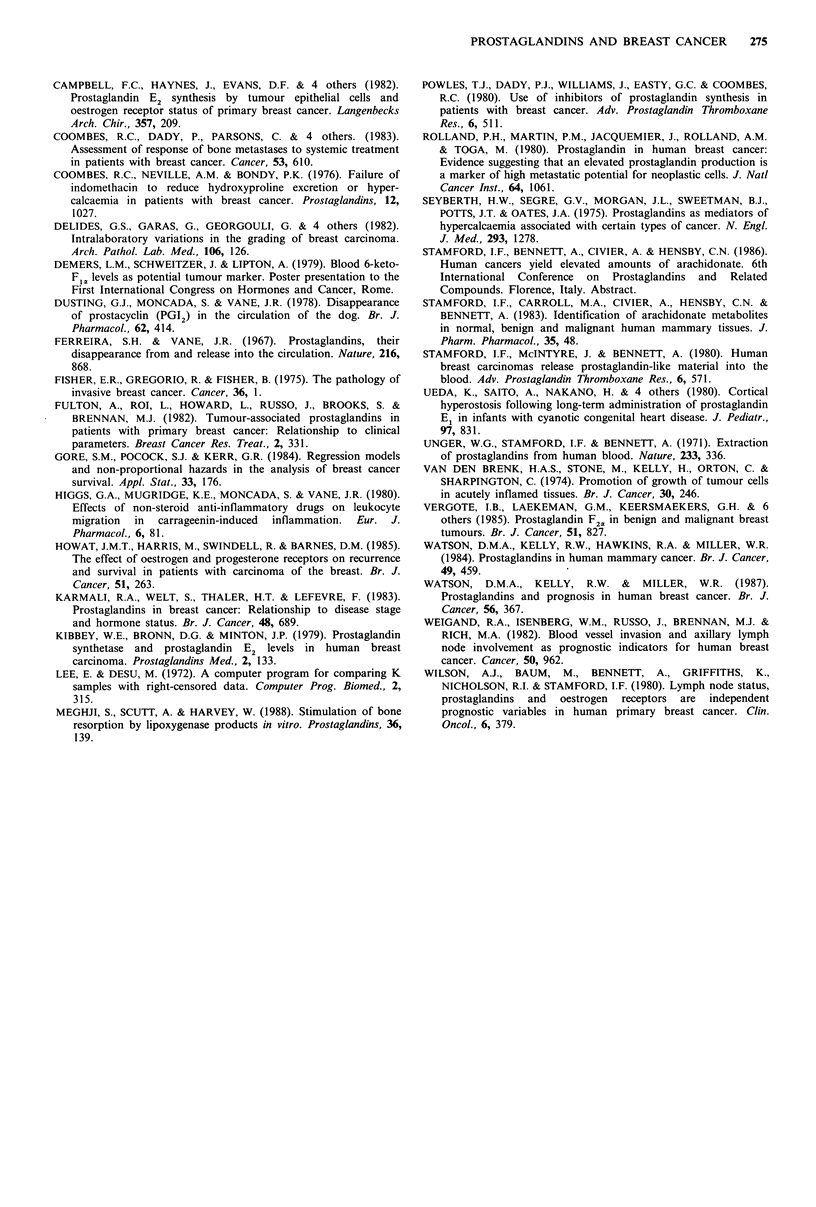


## References

[OCR_01264] BLOOM H. J., RICHARDSON W. W. (1957). Histological grading and prognosis in breast cancer; a study of 1409 cases of which 359 have been followed for 15 years.. Br J Cancer.

[OCR_01243] Bennett A., Charlier E. M., McDonald A. M., Simpson J. S., Stamford I. F., Zebro T. (1977). Prostaglandins and breast cancer.. Lancet.

[OCR_01248] Bennett A., Jarosik C., Sanger G. J., Wilson D. E. (1980). Antagonism of prostanoid-induced contractions of rat gastric fundus muscle by SC-19220, sodium meclofenamate, indomethacin or trimethoquinol.. Br J Pharmacol.

[OCR_01254] Bennett A., McDonald A. M., Simpson J. S., Stamford I. F. (1975). Breast cancer, prostaglandins, and bone metastases.. Lancet.

[OCR_01259] Bennett A., Stamford I. F., Unger W. G. (1973). Prostaglandin E2 and gastric acid secretion in man.. J Physiol.

[OCR_01269] Borah V., Shah P. N., Ghosh S. N., Sampat M. B., Jussawalla D. J. (1980). Further studies on the prognostic importance of Barr body frequency in human breast cancer: with discussion on its probable mechanism.. J Surg Oncol.

[OCR_01287] Coombes R. C., Dady P., Parsons C., McCready V. R., Ford H. T., Gazet J. C., Powles T. J. (1983). Assessment of response of bone metastases to systemic treatment in patients with breast cancer.. Cancer.

[OCR_01292] Coombes R. C., Neville A. M., Bondy P. K., Powles T. J. (1976). Failure of indomethacin to reduce hypercalcemia in patients with breast cancer.. Prostaglandins.

[OCR_01298] Delides G. S., Garas G., Georgouli G., Jiortziotis D., Lecca J., Liva T., Elemenoglou J. (1982). Intralaboratory variations in the grading of breast carcinoma.. Arch Pathol Lab Med.

[OCR_01313] Ferreira S. H., Vane J. R. (1967). Prostaglandins: their disappearance from and release into the circulation.. Nature.

[OCR_01318] Fisher E. R., Gregorio R. M., Fisher B., Redmond C., Vellios F., Sommers S. C. (1975). The pathology of invasive breast cancer. A syllabus derived from findings of the National Surgical Adjuvant Breast Project (protocol no. 4).. Cancer.

[OCR_01333] Higgs G. A., Eakins K. E., Mugridge K. G., Moncada S., Vane J. R. (1980). The effects of non-steroid anti-inflammatory drugs on leukocyte migration in carrageenin-induced inflammation.. Eur J Pharmacol.

[OCR_01339] Howat J. M., Harris M., Swindell R., Barnes D. M. (1985). The effect of oestrogen and progesterone receptors on recurrence and survival in patients with carcinoma of the breast.. Br J Cancer.

[OCR_01345] Karmali R. A., Welt S., Thaler H. T., Lefevre F. (1983). Prostaglandins in breast cancer: relationship to disease stage and hormone status.. Br J Cancer.

[OCR_01350] Kibbey W. E., Bronn D. G., Minton J. P. (1979). Prostaglandin synthetase and prostaglandin E2 levels in human breast carcinoma.. Prostaglandins Med.

[OCR_01355] Lee E. T., Desu M. M. (1972). A computer program for comparing K samples with right-censored data.. Comput Programs Biomed.

[OCR_01360] Meghji S., Sandy J. R., Scutt A. M., Harvey W., Harris M. (1988). Stimulation of bone resorption by lipoxygenase metabolites of arachidonic acid.. Prostaglandins.

[OCR_01365] Powles T. J., Dady P. J., Williams J., Easty G. C., Coombes R. C. (1980). Use of inhibitors of prostaglandin synthesis in patients with breast cancer.. Adv Prostaglandin Thromboxane Res.

[OCR_01371] Rolland P. H., Martin P. M., Jacquemier J., Rolland A. M., Toga M. (1980). Prostaglandin in human breast cancer: Evidence suggesting that an elevated prostaglandin production is a marker of high metastatic potential for neoplastic cells.. J Natl Cancer Inst.

[OCR_01378] Seyberth H. W., Segre G. V., Morgan J. L., Sweetman B. J., Potts J. T., Oates J. A. (1975). Prostaglandins as mediators of hypercalcemia associated with certain types of cancer.. N Engl J Med.

[OCR_01390] Stamford I. F., Carroll M. A., Civier A., Hensby C. N., Bennett A. (1983). Identification of arachidonate metabolites in normal, benign and malignant human mammary tissues.. J Pharm Pharmacol.

[OCR_01396] Stamford I. F., MacIntyre J., Bennett A. (1980). Human breast carcinomas release prostaglandin-like material into the blood.. Adv Prostaglandin Thromboxane Res.

[OCR_01407] Unger W. G., Stamford I. F., Bennett A. (1971). Extraction of prostaglandins from human blood.. Nature.

[OCR_01411] Van Den Brenk H. A., Stone M., Kelly H., Orton C., Sharpington C. (1974). Promotion of growth of tumour cells in acutely inflamed tissues.. Br J Cancer.

[OCR_01416] Vergote I. B., Laekeman G. M., Keersmaekers G. H., Uyttenbroeck F. L., Vanderheyden J. S., Albertyn G. P., Haensch C. F., De Roy G. J., Herman A. G. (1985). Prostaglandin F2 alpha in benign and malignant breast tumours.. Br J Cancer.

[OCR_01421] Watson D. M., Kelly R. W., Hawkins R. A., Miller W. R. (1984). Prostaglandins in human mammary cancer.. Br J Cancer.

[OCR_01426] Watson D. M., Kelly R. W., Miller W. R. (1987). Prostaglandins and prognosis in human breast cancer.. Br J Cancer.

[OCR_01431] Weigand R. A., Isenberg W. M., Russo J., Brennan M. J., Rich M. A. (1982). Blood vessel invasion and axillary lymph node involvement as prognostic indicators for human breast cancer.. Cancer.

